# In Vivo and In Vitro Interactions between Exopolysaccharides from *Bacillus thuringensis* HD270 and Vip3Aa11 Protein

**DOI:** 10.3390/toxins16050215

**Published:** 2024-05-07

**Authors:** Tianjiao Ma, Jinqiu Huang, Pengdan Xu, Changlong Shu, Zeyu Wang, Lili Geng, Jie Zhang

**Affiliations:** 1 College of Life Sciences, Northeast Agricultural University, Harbin 150030, China; tianjiaomippcaas@126.com (T.M.); jinqiuhippcaas@126.com (J.H.); 2 State Key Laboratory for Biology of Plant Diseases and Insect Pests, Institute of Plant Protection, Chinese Academy of Agricultural Sciences, Beijing 100193, China; 3 College of Plant Protection, Anhui Agricultural University, Hefei 230036, China

**Keywords:** *Bacillus thuringiensis*, *Spodoptera frugiperda*, Vip3Aa11, extracellular polysaccharides

## Abstract

*Bacillus thuringiensis* (Bt) secretes the nutritional insecticidal protein Vip3Aa11, which exhibits high toxicity against the fall armyworm (*Spodoptera frugiperda*). The Bt HD270 extracellular polysaccharide (EPS) enhances the toxicity of Vip3Aa11 protoxin against *S. frugiperda* by enhancing the attachment of brush border membrane vesicles (BBMVs). However, how EPS-HD270 interacts with Vip3Aa11 protoxin in vivo and the effect of EPS-HD270 on the toxicity of activated Vip3Aa11 toxin are not yet clear. Our results indicated that there is an interaction between mannose, a monosaccharide that composes EPS-HD270, and Vip3Aa11 protoxin, with a dissociation constant of *Kd* = 16.75 ± 0.95 mmol/L. When EPS-HD270 and Vip3Aa11 protoxin were simultaneously fed to third-instar larvae, laser confocal microscopy observations revealed the co-localization of the two compounds near the midgut wall, which aggravated the damage to BBMVs. EPS-HD270 did not have a synergistic insecticidal effect on the activated Vip3Aa11 protein against *S. frugiperda*. The activated Vip3Aa11 toxin demonstrated a significantly reduced binding capacity (548.73 ± 82.87 nmol/L) towards EPS-HD270 in comparison to the protoxin (34.96 ± 9.00 nmol/L). Furthermore, this activation diminished the affinity of EPS-HD270 for BBMVs. This study provides important evidence for further elucidating the synergistic insecticidal mechanism between extracellular polysaccharides and Vip3Aa11 protein both in vivo and in vitro.

## 1. Introduction

The insect pathogenic bacteria *Bacillus thuringiensis* (Bt) produces Vip proteins during the vegetative growth period and synthesizes crystals proteins, including Cry and Cyt proteins during sporulation [1]. The Vip proteins possess a diverse range of insecticidal activities, demonstrating effectiveness against a diverse array of pests, encompassing Lepidopterans, Coleopterans, Hemipterans, and Dipterans [2,3]. The most thoroughly understood members of the Vip protein family are the Vip3 proteins, which have shown high toxicity against a variety of lepidopteran pests, including *S. frugiperda* [4], *Helicoverpa armigera* [5], *Agrotis ipsilon* [6], *Mythimna separata* [7], and *Spodoptera exigua* [8]. Certain Vip3A class proteins, including Vip3Aa, Vip3Ac, Vip3Ae, and Vip3Af, exhibited significant insecticidal activity towards *S. frugiperda* [9,10]. Functional characterization of Vip3Aa reveals the contributions of specific domains to its insecticidal activity. The N-terminal region of the Vip3A protein exhibits a high degree of conservation and is closely related to its insecticidal function, while the C-terminal sequence exhibits high variability, which may be associated with specific interactions between different target insects [11]. Vip3A protein contains five domains, with domains I-III having the ability to specifically bind to receptors [12], while domains IV and V may affect the interaction between the protein and carbohydrates [13]. Jiang et al. found that domain V can bind to the peritrophic matrix through its glycan-binding activity [14]. The N-terminal helix α1 in domain I of Vip3Aa protein plays a crucial role in toxicity and enhancing liposome permeability and also serves as a cleavage site for midgut protease-activated proteins [15,16]. Once activated, the protein binds to specific receptors on brush border membrane vesicles (BBMVs) within the midgut, and then inserts into the midgut epithelial cell membrane. In *S. frugiperda*, several receptors for Vip3 have been identified, including ribosomal S2 protein [17], fibroblast growth factor receptor (FGFR) [18], and scavenger receptor class C protein (SR-C) [19]. This binding trigger pathological phenomena such as cell swelling, leakage of contents, and cell disintegration, resulting in insect mortality [20].

Extracellular polysaccharides (EPSs), which are large molecular weight polymers, are generated and secreted by Bt throughout its development and metabolism [21]. The diverse functions exhibited by EPSs from *Bacillus* species, including emulsification, immunomodulation, heavy metal chelation, and biological flocculation, suggest their potential for commercial value and applications across various industries, such as food, pharmaceuticals, and environmental management [22]. The EPSs from *B. subtilis* LZ13-4 have good antioxidant ability [23], and EPSs secreted by *B. cereus* AR156 can enhance plant resistance to pathogen invasion [24]. However, there has been limited research exploring the EPSs secreted by Bt. The EPSs of the Bt RSK CAS4 strain have been reported to possess antioxidant and anti-cancer potential [25], while the EPS-2 of the IX01 strain can alter the composition of the human gut microbiota, exhibiting probiotic functions [26]. Our previous studies have revealed that 96.5% of strains in the Bt standard strain library are capable of producing EPSs, and the functions of EPSs vary among different strains [27]. The EPSs produced by Bt strain 4F5 have been found to enhance the effectiveness of defense-related enzymes like catalase (CAT) through the activation of the jasmonic acid and ethylene signaling pathways. This process leads to the induction of systemic resistance against *Sclerotinia sclerotiorum* in *Brassica napus* [28]. The studies also found that Bt EPS-HD270 can enhance the toxicity of both 3D-Cry protoxins and Vip3Aa11 protoxin against different lepidopteran pests, which indicated that the synergistic effect is relatively universal [27]. EPS-HD270 is capable of binding to Cry1Ac protoxin and enhance its binding ability to BBMVs of *Plutella xylostella* larvae [27]. Furthermore, EPS-HD270 exhibited binding affinity for Vip3Aa11 protoxin, increasing its stability in the midgut fluids of *S. frugiperda* and *H. armigera*, and enhancing the binding of Vip3Aa11 protoxin to BBMVs [29]. However, the interaction of EPS-HD270 with Vip3Aa11 protoxin in vivo and the effect on the toxicity of the activated Vip3Aa11 toxin remain elusive.

Bt insecticides occupy over 90% of the global market for biological pesticides [30], and compared to chemical insecticides, Bt formulations are characterized by their non-toxicity to humans and environmental friendliness. However, during the fermentation process of Bt preparations, a large amount of fermentation broth is generated, which requires cleaning and treatment before discharge, thus increasing the economic burden on enterprises. The EPSs secreted by Bt exist in the fermentation broth and are ultimately discharged as a waste liquid. Therefore, exploring the synergistic mechanism and applications of Bt EPSs holds immense potential in enhancing the performance of Bt products, reducing production costs, and expanding the market share of Bt products.

The *S. frugiperda* is categorized within the Noctuidae family of Lepidoptera and is characterized by voracious feeding, strong reproduction, rapid migration, and a wide range of host plants. This pest poses a significant threat to 350 species of plants belonging to 76 families, including cotton, rice, corn, sorghum, and peanuts [31]. In China, the control of *S. frugiperda* mainly relies on chemical control, supplemented by biological control, and Bt products have played an important role in its control.

In this study, *S. frugiperda* is used as the target pest to investigate the interaction between Bt EPSs and Vip3Aa11 protoxin in vivo through fluorescent labeling analysis. Furthermore, the impact of Bt EPSs on the insecticidal activity of the activated Vip3Aa11 toxin is analyzed. While elucidating the mechanism of the enhanced toxicity of EPS-HD270, this study also serves as a theoretical foundation for the recovery of extracellular polysaccharides from fermentation broth during the production of Bt formulations and their industrial applications.

## 2. Results

### 2.1. Purification of EPS-HD270 and Vip3Aa11 Protein

**Figure 1:**
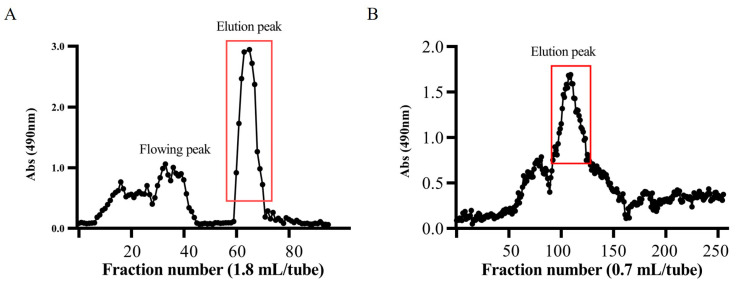
Purification results of EPS-HD270. (**A**) The purification result of EPS-HD270 by anion exchange. (**B**) The purification result of EPS-HD270 by gel filtration chromatography.

The EPS of Bt HD270 strain was extracted through ethanol precipitation. Following proteinase K treatment, SDS-PAGE analysis confirmed the absence of protein residues. Desalting columns were employed to remove small-molecular impurities and sodium chloride from the culture medium. HiTrap Q HP column was further used for purification, and the concentration of EPS in the eluate was detected by the phenol–sulfuric acid method. Results showed that a single extracellular polysaccharide absorption peak appeared when the salt ion concentration was approximately between 30 and 50% (Figure 1A). The corresponding eluate was collected, freeze-dried, and concentrated. Subsequent purification was conducted using a dextran gel chromatography column (Superdex 200 prep grade). Detection with the phenol–sulfuric acid method revealed two distinct EPS absorption peaks (Figure 1B). The EPS corresponding to the peak with the highest absorption was collected, freeze-dried, desalted, and purified to remove NaCl from the elution buffer. This yielded the purified EPS-HD270, and the concentration was 40 mg/mL.

**Figure 2:**
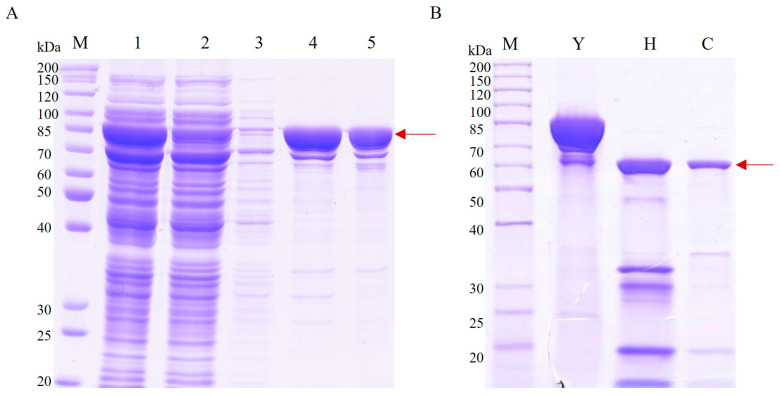
Analysis of the purification and activation results of Vip3Aa11 protoxin by SDS-PAGE. M: Marker 26614; (**A**) 1: Vip3Aa11 soluble expression component; 2: Vip3Aa11 purification process flow through the liquid; 3: binding buffer; 4: elution buffer; and 5: Vip3Aa11 purification result. (**B**) Y: Vip3Aa11 protoxin; H: the Vip3Aa11 trypsin activation result; and C: the purification result of the activated protein using a Q column and a desalting column.

Expression and purification of Vip3Aa11 protoxin were performed under low-temperature conditions. Vip3Aa11 protoxin was primarily expressed in the soluble fraction and was purified using nickel affinity chromatography. Vip3Aa11 protoxin was activated using trypsin, and the activated Vip3Aa11 toxin was purified by utilizing a HiTrap Q HP column and a desalting column. SDS-PAGE results showed that the purified Vip3Aa11 protoxin was a single band of 88 kDa, and the activated Vip3Aa11 toxin was about 65 KDa (Figure 2).

### 2.2. Localization of Vip3Aa11 Protoxin and EPS-HD270 in the Midgut of Spodoptera frugiperda

**Figure 3:**
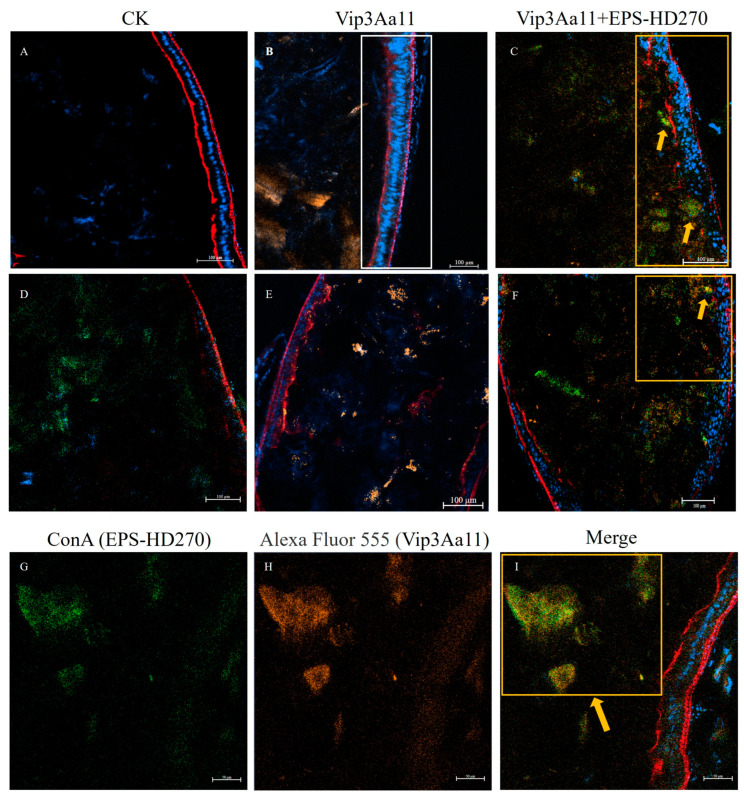
Laser confocal observation of midgut injury and fluorescence signal localization in *S. frugiperda* induced by Vip3Aa11 prototoxin and EPS-HD270; (**A**) PBS buffer feeding control group; (**D**) EPS-HD270 feeding treatment group; (**B**,**E**) Vip3Aa11 protoxin was fed in the treatment group (the white box is the damaged part of intestinal wall cells); (**C**,**F**) the mixture of Vip3Aa11 protoxin and EPS-HD270 was fed in the treatment group (the yellow box line shows the damaged part of intestinal wall cells and the co-localization part of fluorescent signal; the yellow arrows represent regions where fluorescence signal of EPS co-localizes with the Vip3Aa11 protein); (**G**–**I**) the fluorescence images of the mixture of Vip3Aa11 protoxin and EPS-HD270 under different excitation light; (**G**) the green fluorescence of ConA stained EPS-HD270; (**H**) the orange fluorescence of Alexa Fluor 555-labeled Vip3Aa11 protoxin; and (**I**) the merged mixed images of each channel.

Xue et al. found that EPS-HD270 significantly increased the insecticidal activity of Vip3Aa11 protoxin against *S. frugiperda* [29]. To analyze the interaction of EPS-HD270 with Vip3Aa11 protoxin in the midgut of *S. frugiperda*, Alexa Fluor 555-labeled Vip3Aa11 protoxin was mixed with EPS-HD270 and fed to the third-instar larvae for 24 h. The EPS was stained with Concanavalin A fluorescein conjugate fluorescent dye. Alexa Fluor 647 and Hoechst were used to stain the cytoskeleton and cell nuclei of the midgut, respectively. The aggregation and distribution of Vip3Aa11 protoxin and EPS-HD270 in the midgut of *S. frugiperda* were observed using confocal laser scanning microscopy. In the control group fed with buffer alone or EPS-HD270 alone, the epithelial cells in the midgut exhibited intact morphology, with a continuous and undisturbed cytoskeleton and distinct blue-stained cell nuclei (Figure 3A,D). However, in the treatment group fed with only Vip3Aa11 protoxin, the intestinal wall cells showed some degree of damage, with enlarged and elongated cellular cavities (Figure 3B,E). The *rho* and *tau* values of Vip3Aa11 protoxin and EPS-HD270 were 0.741 and 0.631, respectively, indicating a strong positive correlation. In addition, M1 and M2 were 0.960 and 0.791, respectively, indicating a significant degree of co-occurrence between EPS-HD270 and Vip3Aa11 protoxin (Figure 3I). There was a co-localization of Vip3Aa11 protoxin and EPS-HD270 in the vicinity of the intestinal wall cells (Figure 3C,F,I). These observations suggest that the addition of EPS-HD270 exacerbated the damage caused by Vip3Aa11 protoxin in the midgut, further corroborating the synergistic enhancement effect of EPS-HD270 on the insecticidal process of Vip3Aa11 protoxin.

### 2.3. Analysis of Vip3Aa11 Protoxin and Binding to Monosaccharides Contained in EPS-HD270

**Figure 4:**
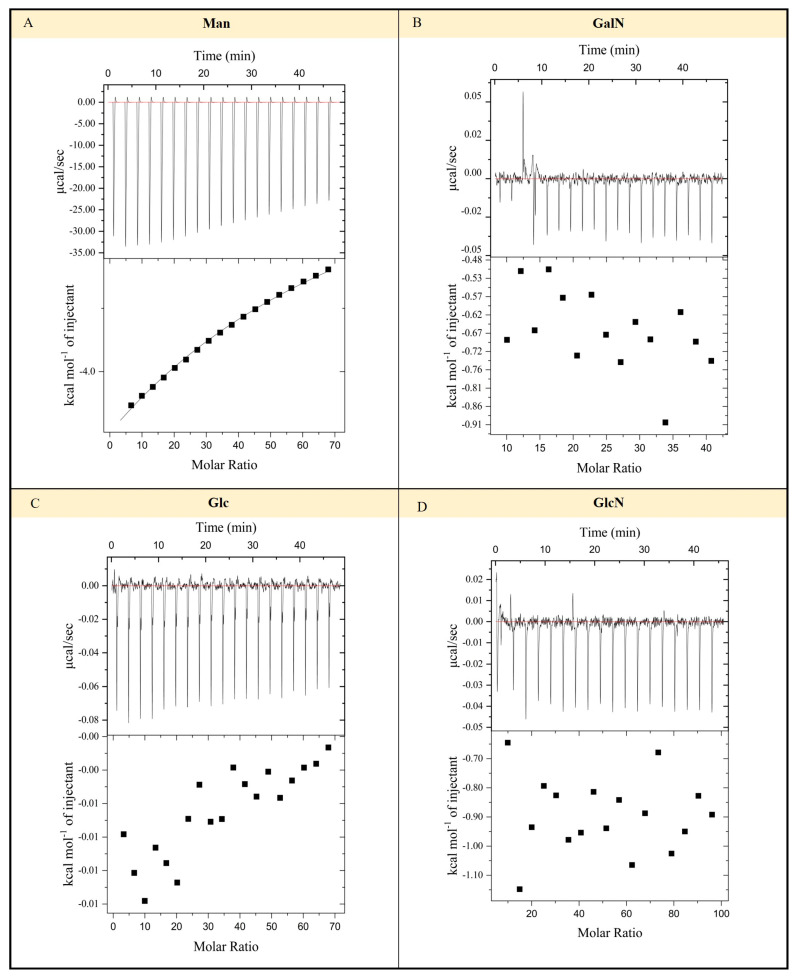
Determination of Vip3Aa11 and carbohydrate binding by isothermal titration calorimetry. ITC reaction curves of Vip3Aa11 protoxin with mannose (**A**), galactose (**B**), glucose (**C**), and glucosamine (**D**).

Previous studies have found that the monosaccharide components of EPS-HD270 mainly include mannose (44.2%), glucosamine (35.5%), galactosamine (8.0%), and glucose (5.5%) [18]. To investigate the binding capacity of Vip3Aa11 protoxin with the four major monosaccharides that compose EPS-HD270, isothermal titration calorimetry (ITC) was used to determine the thermodynamic changes in the reactions between Vip3Aa11 protoxin and different sugars. The ITC results showed that, when mannose was gradually added to the reaction chamber containing Vip3Aa11 protoxin, the interaction between them promoted the formation of a complex, resulting in a detectable exothermic reaction. As the reaction progressed and more mannose was titrated, the thermodynamic value of ITC gradually decreased and stabilized (Figure 4A). However, when galactosamine, glucose, and glucosamine were used as ligands to react with Vip3Aa11 protoxin, the thermodynamic values of ITC were chaotic and could not be fitted into a curve (Figure 4B–D). These results suggest that mannose can bind to Vip3Aa11 protoxin with a dissociation constant (*Kd*) of 16.75 ± 0.95 mmol/L, while galactosamine, glucose, and glucosamine do not interact with Vip3Aa11 protoxin.

### 2.4. Effect of EPS-HD270 on the Insecticidal Activity of the Activated Vip3Aa11 Toxin

**Figure 5:**
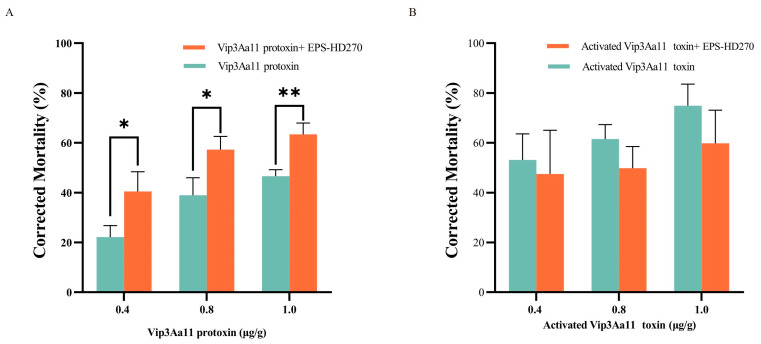
Effects of EPS-HD270 on insecticidal activity of Vip3Aa11 protoxin (**A**) and the activated Vip3Aa11 toxin (**B**) against *S. frugiperda* (7 days). The presented data represent the mean ± standard deviation derived from three separate experiments. Statistical significance is indicated by asterisks (** *p* < 0.01 and * *p* < 0.05).

Previous studies have demonstrated that EPS-HD270 can enhance the toxicity of Vip3Aa11 protoxin [29]. In this study, we further explored the impact of EPS-HD270 on the toxicity of the activated Vip3Aa11 toxin. It was found that, when the concentrations of Vip3Aa11 protoxin were 0.4, 0.8, and 1.0 μg/g, the addition of EPS-HD270 at a final concentration of 0.5 mg/g substantially elevated the corrected mortality rate of *S. frugiperda* larvae from 22.16%, 38.95%, and 46.57% to 40.47% (*p* < 0.05), 57.26% (*p* < 0.05), and 63.37% (*p* < 0.01), respectively (Figure 5A). However, when the same concentrations of Vip3Aa11 toxin were fed to *S. frugiperda* larvae upon incorporating EPS-HD270, the mortality of the larvae after 7 days was 47.48%, 49.80%, and 59.84%, respectively, which did not exhibit a significant difference compared with the treatment group lacking EPS supplementation (*p* > 0.05) (Figure 5B). This indicates that the addition of EPS has no synergistic effect on the toxicity of the activated Vip3Aa11 toxin.

### 2.5. The Interaction of EPS-HD270 with Vip3Aa11 Protoxin and the Activated Toxin

**Figure 6:**
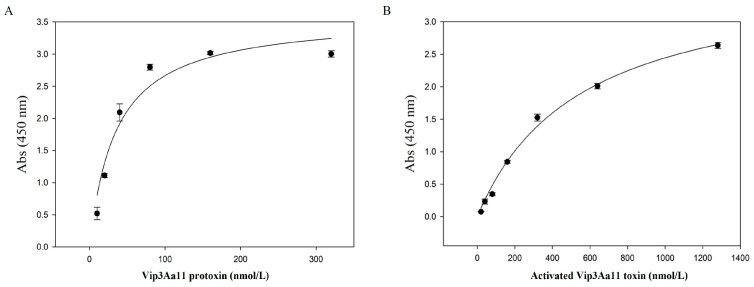
ELISA analyzed the interaction between EPS-HD270 and Vip3Aa11 protoxin (**A**) and the activated Vip3Aa11 toxin (**B**).

Previous studies have shown that EPS-HD270 can bind to Vip3Aa11 protoxin [20]. In this study, the binding ability of EPS-HD270 to the activated Vip3Aa11 toxin was further analyzed using ELISA. The results showed that the activated Vip3Aa11 toxin can bind to EPS-HD270 in a saturated manner. The dissociation constant (*Kd*) was calculated to be 548.73 ± 82.87 nmol/L. While the *Kd* of the same concentration of extracellular polysaccharide with Vip3Aa11 protoxin was 34.96 ± 9.00 nmol/L (Figure 6). Therefore, the activated Vip3Aa11 toxin can bind to EPS-HD270, but its binding ability is significantly lower than that of protoxin with EPS.

### 2.6. Analysis of the Effect of EPS-HD270 on the Association of Activated Vip3Aa11 Protein with the BBMV of S. frugiperda

**Figure 7:**
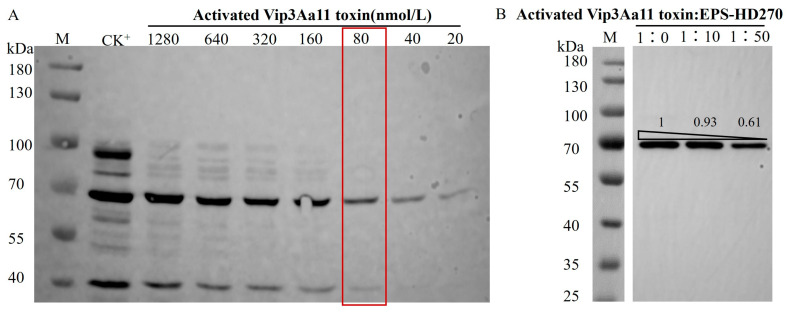
Influence of EPS-HD270 on the interaction of the activated Vip3Aa11 toxin with BBMVs of *S. frugiperda*. M: Marker 26616. (**A**) The binding analysis of Vip3Aa11 toxin and BBMVs, and CK^+^ shows the saturated bound Vip3Aa11 protoxin and BBMVs. (**B**) The impact of EPS-HD270 on the interaction between the activated Vip3Aa11 toxin and BBMVs. Furthermore, 1:0, 1:10, 1:50 and 1:100 are the mass ratios of activated Vip3Aa11 toxin to EPS-HD270.

The research conducted by Xue et al. demonstrated that EPS-HD270 potentiates the binding capacity of Vip3Aa11 protoxin to the BBMVs of *S. frugiperda* larvae [29]. In this study, we further delved into the impact of EPS-HD270 on the interaction between the activated Vip3Aa11 protein and BBMVs. Western blot results indicated that, when the concentration of the activated Vip3Aa11 protein was 80 nmol/L, the binding to BBMVs was unsaturated (Figure 7A). At this concentration of the activated Vip3Aa11 protein, EPS-HD270 was added at a mass ratio of 1:10 and 1:50, respectively, and incubated with 20 μg of BBMVs from *S. frugiperda* larvae. Western blot results (Figure 7B) revealed that as the mass of added EPS-HD270 increased, the binding capacity of the activated Vip3Aa11 protein to BBMVs decreased.

## 3. Discussion

Wang et al. reported that 96.5% of Bt standard strains can produce EPSs [27]. EPS-HD270 was discovered to potentiate the toxicity of Cry1Ac protoxin towards *P. xylostella*, but it exhibited no synergistic influence on the toxicity of the activated Cry1Ac toxin [27]. Xue et al. reported that EPS-HD270 significantly improves the toxicity of Vip3Aa11 protoxin against *S. frugiperda* and *Helicoverpa armigera* larvae [29]. In this study, Vip3Aa11 protoxin was activated with trypsin, and the findings indicated that EPS-HD270 did not exhibit a collaborative enhancement in the toxicity of the activated Vip3Aa11 toxin.

EPS-HD270 was discovered to associate with Vip3Aa11 protoxin, enhance its binding to BBMVs and stability in midgut fluid [29]. In this study, laser confocal microscopy results revealed a co-localization of fluorescence signals between EPS-HD270 and Vip3Aa11 protoxin in the midgut, accompanied by increased midgut damage in *S. frugiperda* larvae. The midgut cells of *S. frugiperda* fed with both Vip3Aa11 protoxin and EPS-HD270 exhibited signs of disorganization and were freely dispersed within the intestinal lumen. The cytoskeleton of the intestinal wall sustained severe damage. These findings suggest that Vip3Aa11 protoxin interacts with EPS-HD270, thereby potentiating its toxicity in vivo.

Notably, this study found that the binding capacity of EPS-HD270 with activated Vip3Aa11 toxin is lower than its binding capacity with Vip3Aa11 protoxin, and it also reduces the binding capacity of activated Vip3Aa11 toxin to BBMVs of *S. frugiperda* larvae. This is consistent with our previous study where EPS-HD270 decreases the binding of activated Cry1Ac to BBMVs of *P. xylostella* [27]. The reduction in toxicity may be due to the decreased binding ability of the activated Vip3Aa11 toxin to BBMVs after the addition of EPS. Furthermore, as the concentration of EPS elevates, the interaction between activated Vip3Aa11 or Cry1Ac protein [27] and BBMVs diminishes progressively, indicating that EPS may compete with activated proteins for binding sites on BBMVs. Currently, there is uncertainty regarding whether the activation of Vip3Aa11 protoxin precedes or follows receptor binding, along with the identification of the receptor protein [32]. It is known that, compared to the protoxin, the activated toxin exhibits important conformational changes in the helical structure of the N-terminal region, while the three C-terminal domains III-V remain unchanged. Domains IV and V are both glycan-binding motifs [33]. However, the role of domains IV and V in the interaction between the activated toxin and EPS, as well as the impact of conformational alterations in domains I and II on the reduced binding capacity of EPS to the protein, remains unclear. Furthermore, ITC experiments have demonstrated an interaction between mannose, one of the major monosaccharides present in EPS-HD270, and Vip3Aa11 protoxin. No interaction was observed with glucosamine, glucose, or galactose. Further analysis is necessary to clarify the binding sites between different domains and EPS, as well as the binding sites between monosaccharide components and protein, in order to comprehend the role EPS plays in the insecticidal process mediated by protein.

Currently, Bt EPSs are discarded as waste during the production process due to their presence in the fermentation broth. Extracting EPS from the fermentation broth reduces the production cost of Bt products and improves their effectiveness. To address this issue, we employed a previously established EPS–protein interaction platform for Bt strains to analyze the interaction between Vip3Aa11 protoxin and EPS. Through both in vitro binding assays and in vivo observations, we investigated the interactions between Vip3Aa11 protein and EPS-HD270. The findings of this study contribute valuable insights into elucidating the mechanism by which EPS modulates the toxicity of Vip3Aa11 protoxin against lepidopteran pests. These revelations provide a crucial theoretical framework for the industrial exploitation of bacterial EPS within the fermentation broth during the production of *Bacillus thuringiensis* (Bt).

## 4. Conclusions

In this study, it was found that EPS-HD270 aggravated Vip3Aa11 protoxin-induced midgut injury, and the co-localization of EPS-HD270 and Vip3Aa11 was observed in the midgut in vivo. Mannose interacted with Vip3Aa11 protoxin, but no interaction was found with glucosamine, glucose, and galactose. EPS-HD270 had no synergistic effect with the activated Vip3Aa11 toxin, and the binding ability of EPS-HD270 to activated Vip3Aa11 toxin was lower than that to protoxin. EPS-HD270 attenuated the interaction between the activated Vip3Aa11 toxin and BBMVs of *S. frugiperda*. The above results helped to clarify the mechanism by which EPSs influence the insecticidal activity of Vip3Aa11 protoxin against lepidopteran pests.

## 5. Materials and Methods

### 5.1. Strains and Insect Culture

The extracellular polysaccharides were extracted from the Bt serovar *kurstaki* HD270 strain. The *vip3Aa11* gene was carried by the plasmid pET28a. All strains were maintained in our laboratory. The cultivation temperature for the Bt strain was 30 °C, while for *Escherichia coli*, it was 37 °C. The culture medium used was LB liquid medium.

In this study, *Spodoptera frugiperda* larvae were cultured in our laboratory. The formula for the artificial diet was provided by the cotton pest research group of the same institute. The larvae were reared under conditions of (27 ± 1) °C, (65 ± 5)% relative humidity, and a photoperiod of 14L:10D.

### 5.2. Expression and Purification of Insecticidal Protein

Vip3Aa11 protoxin was induced for expression at a low temperature of 18 °C following the experimental methods of Wang et al. [34]. The protein was then extracted and purified using a nickel affinity column to remove contaminating proteins, followed by desalting using HiPrepTM26/10 Desalting (GE Healthcare Life Sciences, Chicago, IL, USA). Vip3Aa11 protoxin was activated by incubation with trypsin at a mass ratio of 50:1 (protoxin/trypsin) at 37 °C. After activation, the protein was purified using a HiTrap Q column (GE Healthcare Life Sciences, Chicago, IL, USA) and a desalting column. The protein peak and Cond peak were monitored during the collection procedure to make sure that the protein peak was collected before the Cond value increased.

### 5.3. Extraction and Purification of EPS-HD270

The cultivation of the strain and the extraction and purification of exopolysaccharides were carried out following the experimental methods described by Wang et al. [27]. The fermentation supernatant of the Bt HD270 strain was precipitated overnight by mixing with three times the volume of 95% ethanol. The precipitate was then centrifuged and resuspended in ultrapure water. The quantity of protein in the blend was measured utilizing the BCA assay kit (Solarbio Life Sciences, Beijing, China), and protease K (Merck, Darmstadt, Germany) was incorporated into the mixture at a concentration of 10% relative to the protein weight. The mixture was incubated at 50 °C for 2 h, followed by boiling at 100 °C for 20 min to deactivate protease K, ultimately yielding a crude exopolysaccharide solution.

The crude exopolysaccharide solution was purified using the AKTA avant 150 system. Low-molecular-weight impurities were eliminated via a desalting column, followed by the purification of the target polysaccharide using the HiTrap Q HP column. Target EPSs were collected and freeze-dried for concentration. HiLoad 26/600 Superdex 200 (GE Healthcare Life Sciences, Chicago, IL, USA) was employed to further separate and purify the main component. The exopolysaccharides were finally desalted, and their concentration was quantified.

### 5.4. Monosaccharides Bind to Vip3Aa11 Protoxin

To analyze the interaction between Vip3Aa11 protoxin and monosaccharides, ITC was employed using a MicroCal iTC200 instrument (GE Healthcare Life Sciences, Chicago, IL, USA). Both the protein and monosaccharide buffers were exchanged with a 20 mmol/L Tris-HCl solution (pH 8.0). The titration experiments were performed at 25 °C with 19 injections of 50 mmol/L monosaccharides into a cell containing 150 μmol/L protein (~200 μL). The samples were stirred at a speed of 1000 rpm. Each injection was 2 μL and lasted for 4 s, with a 150 s interval between injections. The heat of titration of each monosaccharide into the buffer under identical conditions was used as a control for subtraction.

### 5.5. Localization of Vip3Aa11 and EPS-HD270 in Spodoptera frugiperda

Third-instar larvae of *S. frugiperda* were fed with a mixture of Alexa Fluor 555 (Thermo Fisher Scientific, Waltham, MA, USA)-labeled Vip3Aa11 protoxin and EPS-HD270 for 24 h. Intact midguts were dissected and fixed overnight at 4 °C in a cell tissue fixative solution in darkness. The midguts were washed three times with PBS buffer, stained with Alexa Fluor 647 (Thermo Fisher Scientific, Waltham, MA, USA) (diluted in PBS with a ratio of 1:1000) for 50 min, Hoechst 33,342 (Solarbio Life Sciences, Beijing, China) (diluted in PBS with a ratio of 1:100) for 10 min, and Concanavalin A Conjugates (Thermo Fisher Scientific, Waltham, MA, USA) (buffered with 0.1 mol/L NaHCO_3_) for 30 min. The dye was removed, and the midguts were then flushed three times with PBS for 5 min each time. A drop of anti-fluorescence quenching mounting medium was applied to the center of the recess on the concave slide, and three midguts were placed in each recess. The accumulation and distribution of Vip3Aa11 protoxin and EPS in the midguts of *S. frugiperda* were visualized through a confocal laser scanning microscope (ZEISS LSM 980).

### 5.6. Preparation of BBMVs

The larvae of *S. frugiperda* were reared until they reached the third-instar stage, and their midguts were subsequently dissected. BBMVs were then prepared utilizing the magnesium precipitation method [25]. The purity of the BBMVs was assessed by determining the aminopeptidase N (APN) activity.

### 5.7. Bioassay

Bioassay was conducted as described in Xue et al. [29]. To determine the insecticidal activity of the activated Vip3Aa11 toxin, EPS, and their mixture against the first-instar larvae of *S. frugiperda*, we conducted bioassays. The artificial diets were dispensed into each Petri dish and 3 mL of the protein–EPS mixture was incorporated into the diet. After evaporation of the moisture, the diet was evenly distributed into a 24-well plate. The first-instar larvae were then introduced into each well and covered with paper towels. Each treatment was replicated three times, with 24 larvae per replicate. The Vip3Aa11 buffer (20 mmol/L Tris-HCl) and EPS buffer (ultra-pure water) were used as controls in the same proportions. The larvae and diet were observed daily, and the corrected mortality was calculated after 7 days.

### 5.8. ELISA Assay of the Binding Ability of Vip3Aa11 Protoxin and Activated Toxin to EPS-HD270

ELISA was conducted as described in Xue et al. [29]. A volume of 100 µL containing 15 mg/mL of EPS-HD270 was added to a 96-well ELISA plate (Nunc Maxisorp, Thermo Fisher Scientific, Waltham, MA, USA) and incubated at 4 °C overnight. After blocking, different concentrations of Vip3Aa11 protoxin (0, 10, 20, 40, 80, 160, 320 nmol/L) or the activated Vip3Aa11 toxin (0, 20, 40, 80, 160, 320, 640, 1280 nmol/L) were added and incubated at 37 °C for 1 h. The cells were incubated with Vip3Aa11 antibody (diluted 1:5000 in TBST, 100 μL) and horseradish peroxidase (HRP)-labeled goat anti-mouse IgG antibody (diluted 1:10,000 in TBST, 100 μL) for 1 h at 37 °C. After each incubation, the cells were washed three times with TBST for 10 min each time. A 100 µL volume of TMB solution (Solarbio Life Sciences, Beijing, China) was used for the reaction in a darkened environment at 37 °C for a period of 20 min. The reaction was halted through the addition of 100 µL HCl (2.0 mol/L), and the Abs 450 nm was quantified using a microplate reader.

### 5.9. Western Blot Analysis of the Binding of Activated Vip3Aa11 toxin with EPS-HD270 to BBMVs of S. frugiperda

To investigate the influence of EPS-HD270 on the interaction between the activated Vip3Aa11 toxin and BBMVs, we employed the experimental method mentioned by Xue et al. [29]. We conducted a binding assay using various concentrations of activated Vip3Aa11 toxin with BBMVs of *S. frugiperda*. Unsaturated binding concentrations of protein were selected and incubated overnight at 4 °C with different mass ratios (1:0, 1:10, 1:50) of EPS-HD270 and 20 μg of BBMV. The mixture underwent centrifugation at 4 °C for 30 min at a speed of 18,000× *g*. The resulting precipitates were then prepared as samples for Western blot analysis.

The PVDF membrane was blocked with 5% skim milk powder for 1 h at 60 r/min. The blocked PVDF membrane was incubated with antibodies (the antibody dilution was the same as the ELISA experiment, and the incubation condition was 60 r/min for 1 h at room temperature). After incubation with Vip3Aa11 antibody and horseradish peroxidase (HRP)-labeled goat anti-mouse IgG antibody, the PVDF membrane was washed three times with TBST for 10 min each time. After applying SuperSignalTM West Pico PLUS chemiluminescent substrate (Thermo Fisher Scientific, Waltham, MA, USA) to the membrane, an e-BLOT Touch Imager was used for detection.

### 5.10. Data Analysis

Statistical significance was analyzed using one-way ANOVA test with SPSS (IBM, Armonk, NY, USA). Dissociation constants (*Kd*) were calculated using the Sigma-plot v.14.0 software. Grayscale quantitation of protein bands was performed using Image J. Multiple co-localization coefficients, Pearson (r), Spearman (ρ, rho), Kendall (τb, tau), and Manders (M1, M2), coefficients were calculated using the Image J Coloc 2 plugin to assess the degree of co-localization [35,36]. The statistical analysis depicted in the figure was carried out using GraphPad Prism (version 8.0). Raw data from ITC experiments were processed and analyzed using Origin 7.0 software.

## Data Availability

No new sequencing data were created or analyzed in this study.
